# An Electroelastic Solution for Functionally Graded Piezoelectric Circular Plates under the Action of Combined Mechanical Loads

**DOI:** 10.3390/ma11071168

**Published:** 2018-07-09

**Authors:** Zhi-xin Yang, Xiao-ting He, Xue Li, Yong-sheng Lian, Jun-yi Sun

**Affiliations:** 1 School of Civil Engineering, Chongqing University, Chongqing 400045, China; yangzhixin123@126.com (Z.-x.Y.); lixuecqu@126.com (X.L.); lianyongsheng@cqu.edu.cn (Y.-s.L.); sunjunyi@cqu.edu.cn (J.-y.S.); 2 Key Laboratory of New Technology for Construction of Cities in Mountain Area (Chongqing University), Ministry of Education, Chongqing 400045, China

**Keywords:** functionally graded piezoelectric materials, circular plate, combined mechanical loads, electroelastic solution

## Abstract

In this study, we obtained an electroelastic solution for functionally graded piezoelectric circular plates under the action of combined mechanical loads which include the uniformly distributed loads on the upper surface of the plate and the radial force and bending moment at the periphery of the plate. All electroelastic materials parameters are assumed to vary according to the same gradient function along the thickness direction. The influence of different functionally graded parameters on the elastic displacement and elastic stress, as well as the electric displacement and electric potential, was discussed by a numerical example. The solution presented in this study is not only applicable to the case of combined loads, but also to the case of a single mechanical load. In addition, this solution reflects the influence of the function gradient on the pure piezoelectric plate, which is helpful to the refined analysis and optimization design of similar structures.

## 1. Introduction

The concept of functionally graded materials (FGMs) can be traced back to the eighties and nineties of last century, and at that time, to eliminate interface problems and relieve thermal stress concentrations in conventional laminated materials, a group of Japanese scientists suggested using this material as thermal barrier materials for aerospace structural applications and fusion reactors [1]. Generally, FGMs are a kind of inhomogeneous composite from the point of macroscopic view that are typically made from a mixture of two materials. This mixture can be obtained by gradually changing the composition of the constituent materials (along the thickness direction of components in most cases). The characteristics of FGMs vary gradually with the thickness direction within the structure, which eliminates interface problems, and thus the stress distributions are smooth. Moreover, FGMs possess many new properties that most traditional laminated materials do not have, which gives the use of FGMs many advantages in aerospace, automotive, and biomedical applications. During the past decades, FGMs have received a significant amount of attention from the academic community and engineering field, and many scholars have carried out research on functionally graded materials and structures [2,3,4,5,6,7,8,9,10,11,12].

On the other hand, piezoelectric materials have been used extensively in the design of sensors and actuators due to their high efficiency in electromechanical conversion [13,14,15]. Piezoelectric sensors are usually a laminated original made by ceramic slice. However, on this kind of laminated original, it is easy to cause stress concentration and promote the growth of interfacial microcracks which limit the application and development of the piezoelectric original. In order to solve this problem, functionally graded piezoelectric materials (FGPMs), whose material properties change continuously in one direction, were developed [16,17,18,19]. Because there is no obvious interface in this material, the damage caused by the stress concentration at the interface can be avoided.

With the increasing application of functionally graded piezoelectric materials, precise characterization of their mechanical properties is urgently needed. A great deal of research has been done on the mechanical properties of functionally graded piezoelectric materials. Dineva et al. [20] evaluated the stress and electric field concentrations around a circular hole in a functionally graded piezoelectric plane subjected to antiplane elastic SH-wave and in-plane, time-harmonic electric load. Chen and Ding [21] investigated the bending problem of a simply supported rectangular plate by introducing two displacement functions and stress functions and combining the state space method. Zhang et al. [22] studied the behavior of four parallel nonsymmetric permeable cracks with different lengths in a functionally graded piezoelectric material plane subjected to antiplane shear stress loading by the Schmidt method. Wu et al. [23] analyzed the electromechanical coupling effect for functionally graded piezoelectric plates. The coupled static analysis of thermal power and electricity for functionally graded piezoelectric rectangular plates was carried out by Zhong and Shang [24,25]. Based on the generalized Mindlin plate theory, Zhu et al. [26] derived the finite element equations of functionally graded material plates by using the variation principle and investigated and calculated the deflection and potential of a simply supported functionally graded piezoelectric square plate with linear gradient under uniformly distributed loads. Lu et al. [27,28] studied the bending problem of a simply supported functionally gradient piezoelectric plate and a cylindrical plate under mechanical load separately by using the similar Stroh equation. The exact solution of free vibration of functionally graded piezoelectric circular plates was studied by Zhang and Zhong [29]. Recently, Liu et al. [30] presented transient thermal dynamic analysis of stationary cracks in functionally graded piezoelectric materials based on the extended finite element method (X-FEM). Yu et al. [31] analyzed interfacial dynamic impermeable cracks in dissimilar piezoelectric materials under coupled electromechanical loading with the extended finite element method. Given that there are many studies in this field, here we do not review them in detail.

Among the studies above, we note that since the materials parameters vary with a certain direction and the electromechanical coupling effect exists, the obtainment of an analytical solution is relatively difficult. The basic equations of functionally graded piezoelectric structures are generally expressed in the form of partial differential equations except for the physical equations. The general practice is still the so-called separation of variables. According to the specific problem, for example, a spatial axisymmetric deformation problem in [32,33], the unknown stress or displacement function and the unknown electrical potential function are expressed as a polynomial with respect to two variables, i.e., $F(r,z)=\sum r^{n}f_{n}(z)$, in which *r* is the radial coordinate and *z* is the transverse coordinate along the thickness direction. By continuous substitution and integration, the partial differential equations are transformed into ordinary differential equations, and the integral constants may be determined by boundary conditions, thus obtaining the final solution. Besides, to the authors’ knowledge, the existing work of functionally graded piezoelectric plates focused mostly on the problem of the plate subjected to a single load, and the problem under the action of combined mechanical loads seems to be relatively less.

In this study, we will analyze the axisymmetric deformation problem of functionally graded piezoelectric circular plates under the action of combined mechanical loads (i.e., uniformly distributed loads on the upper surface of the plate and radial force and bending moment at the periphery of the plate). The basic equations and their electroelastic solution are presented in Section 2. In Section 3, the influence of different functionally graded parameters on the elastic displacement and stress, as well as the electric displacement and electric potential, are discussed by a numerical example. Section 4 is the concluding remarks.

## 2. Basic Equations and Their Electroelastic Solution

**Figure 1:**
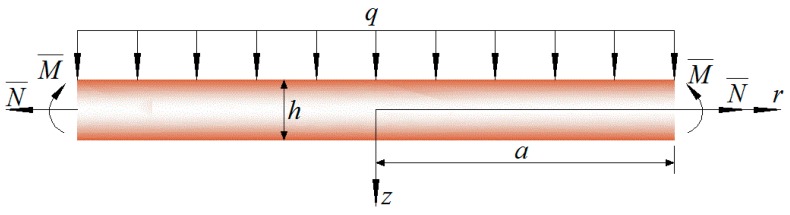
Sketch of a functionally graded piezoelectric circular plate.

Considering a simply supported functionally graded piezoelectric circular plate with radius a and thickness h, a uniformly distributed load q is applied on the upper surface of the plate and a radial force $\bar{N}$ and a bending moment $\bar{M}$ are applied at the periphery of the plate, as shown in Figure 1.

Here, we introduce the cylindrical coordinate system (r,θ,z), where the upper and lower surfaces of the plate are z=−h/2 and z=h/2, respectively, the center of the plate is r=0, and the periphery of the plate is r=a. The physical parameters of functionally graded piezoelectric materials are usually the functions of coordinates, and in many practical situations, the physical parameters change only in one direction. In this study, we assumed that the material parameters vary according to the same function along the thickness direction,

(1) $$c_{ij}=c_{ij}^{0}f(z),e_{ij}=e_{ij}^{0}f(z),\lambda_{ij}=\lambda_{ij}^{0}f(z),$$

in which $f(z)=e^{\alpha z/h}$ is the gradient function, α is the functional gradient parameter, $c_{ij}$, $e_{ij}$, $\lambda_{ij}$ are elastic, piezoelectric, and dielectric parameters, respectively, and $c_{ij}^{0}$, $e_{ij}^{0}$, $\lambda_{ij}^{0}$ are the values of the corresponding material parameters at z=0. Supposing that the polarization direction is the forward direction of the z axis, let us take a microelement in the circular plate, and from the balance of the force, we can obtain

(2) $$\begin{array}{l} \frac{\partial \sigma_{r}}{\partial r}+\frac{\sigma_{r}-\sigma_{\theta }}{r}+\frac{\partial \tau_{zr}}{\partial z}=0\\ \frac{\partial \tau_{rz}}{\partial r}+\frac{\partial \sigma_{z}}{\partial z}+\frac{\tau_{rz}}{r}=0 \end{array}\},$$

in which $\sigma_{r}$ is the radial stress, $\sigma_{\theta }$ is the circumferential stress, $\sigma_{z}$ is the stress in the thickness direction, and $\tau_{rz}$, $\tau_{zr}$ are the tangential stress. The equation of Maxwell electric displacement conservation is

(3) $$\frac{\partial D_{r}}{\partial r}+\frac{\partial D_{z}}{\partial z}+\frac{D_{r}}{r}=0,$$

in which $D_{r}$ and $D_{z}$ are the electric displacement components, respectively. In the cylindrical coordinate system (r,θ,z), the physical equations of transversely isotropic, functionally graded piezoelectric materials with the z axis being normal to the plane of isotropy read

(4) $$\begin{array}{l} \sigma_{r}=c_{11}\varepsilon_{r}+c_{12}\varepsilon_{\theta }+c_{13}\varepsilon_{z}-e_{31}E_{z}\\ \sigma_{\theta }=c_{12}\varepsilon_{r}+c_{11}\varepsilon_{\theta }+c_{13}\varepsilon_{z}-e_{31}E_{z}\\ \sigma_{z}=c_{13}\varepsilon_{r}+c_{13}\varepsilon_{\theta }+c_{33}\varepsilon_{z}-e_{33}E_{z}\\ \tau_{zr}=c_{44}\gamma_{zr}-e_{15}E_{r}\\ D_{r}=e_{15}\gamma_{zr}+\lambda_{11}E_{r}\\ D_{z}=e_{31}(\varepsilon_{r}+\varepsilon_{\theta })+e_{33}\varepsilon_{z}+\lambda_{33}E_{z} \end{array},$$

in which $\varepsilon_{r}$, $\varepsilon_{\theta }$, $\varepsilon_{z}$, $\gamma_{zr}$ are strain components, and $E_{r}$, $E_{z}$ are the electric field in r and z directions, respectively. The geometric equations are

(5) $$\begin{array}{l} \varepsilon_{r}=\frac{\partial u_{r}}{\partial r},\varepsilon_{\theta }=\frac{u_{r}}{r}\\ \varepsilon_{z}=\frac{\partial u_{z}}{\partial z},\gamma_{rz}=\frac{\partial u_{z}}{\partial r}+\frac{\partial u_{r}}{\partial z} \end{array}\},$$

in which $u_{r}$, $u_{z}$ are the displacement in r and z directions, respectively. The relation of electric field and electric potential is

(6) $$E_{r}=-\frac{\partial \phi }{\partial r},E_{z}=-\frac{\partial \phi }{\partial z},$$

in which ϕ is the electric potential. Those equations shown above are the basic equations of the problem presented here. The boundary conditions, which can be used for the solution of those basic equations, are shown as follows:

(7a) $$\sigma_{z}=-q,\tau_{rz}=0,D_{z}=0\;\mathrm{at}\;z=-h/2,$$

(7b) $$\sigma_{z}=0,\tau_{rz}=0,D_{z}=0\;\mathrm{at}\;z=h/2,$$

(7c) $$N(r)=\bar{N},M(r)=\bar{M},u_{z}(r,0)=0,\int_{-h/2}^{h/2}D_{r}dz=0,\tau_{rz}=0\;\mathrm{at}\;r=a.$$

Suppose that [32,33]

(8) $$\begin{array}{l} u_{r}(r,z)=ru_{1}(z)+r^{3}u_{3}(z)\\ u_{z}(r,z)=w_{0}(z)+r^{2}w_{2}(z)+r^{4}w_{4}(z)\\ \phi (r,z)=\phi_{0}(z)+r^{2}\phi_{2}(z)+r^{4}\phi_{4}(z) \end{array}\},$$

in which $u_{i}(z)$ and $w_{i}(z)$ are also looked at as the displacement functions, $\phi_{i}(z)$ is also looked at as the potential functions, and they depend only on *z*. The detailed reason for the assumption of Equation (8) is shown in the Appendix A, which includes some results from functionally graded piezoelectric beams [34,35]. Substituting Equation (8) into Equation (5), it gives

(9) $$\begin{array}{l} \varepsilon_{r}=u_{1}(z)+3r^{2}u_{3}(z),\;\varepsilon_{\theta }=u_{1}(z)+r^{2}u_{3}(z)\\ \varepsilon_{z}=w_{0}^{\prime }(z)+r^{2}w_{2}^{\prime }(z)+r^{4}w_{4}^{\prime }(z),\;\gamma_{rz}=2rw_{2}(z)+4r^{3}w_{4}(z)+ru_{1}^{\prime }(z)+r^{3}u_{3}^{\prime }(z) \end{array}\}.$$

Substituting Equations (6), (8), and (9) into Equation (4), we can obtain

(10) $$\begin{array}{l} \sigma_{r}=[e_{31}\phi_{4}^{\prime }(z)+c_{13}w_{4}^{\prime }(z)]r^{4}+[3c_{11}u_{3}(z)+c_{12}u_{3}(z)+c_{13}w_{2}^{\prime }(z)+e_{31}\phi_{2}^{\prime }(z)]r^{2}\\ \;+[c_{11}u_{1}(z)+c_{12}u_{1}(z)+c_{13}w_{0}^{\prime }(z)+e_{31}\phi_{0}^{\prime }(z)]\\ \sigma_{\theta }=[c_{13}w_{4}^{\prime }(z)+e_{31}\phi_{4}^{\prime }(z)]r^{4}+[3c_{12}u_{3}(z)+c_{11}u_{3}(z)+c_{13}w_{2}^{\prime }(z)+e_{31}\phi_{2}^{\prime }(z)]r^{2}\\ \;+[c_{12}u_{1}(z)+c_{11}u_{1}(z)+c_{13}w_{0}^{\prime }(z)+e_{31}\phi_{0}^{\prime }(z)]\\ \sigma_{z}=[c_{33}w_{4}^{\prime }(z)+e_{33}\phi_{4}^{\prime }(z)]r^{4}+[4c_{13}u_{3}(z)+c_{33}w_{2}^{\prime }(z)+e_{33}\phi_{2}^{\prime }(z)]r^{2}\\ \;+[2c_{13}u_{1}(z)+c_{33}w_{0}^{\prime }(z)+e_{33}\phi_{0}^{\prime }(z)]\\ \tau_{zr}=[2c_{44}w_{2}(z)+c_{44}u_{1}^{\prime }(z)+2e_{15}\phi_{2}(z)]r+[4c_{44}w_{4}(z)+c_{44}u_{3}^{\prime }(z)+4e_{15}\phi_{4}(z)]r^{3}\\ D_{r}=[2e_{15}w_{2}(z)+e_{15}u_{1}^{\prime }(z)-2\lambda_{11}\phi_{2}(z)]r+[4e_{15}w_{4}(z)+e_{15}u_{3}^{\prime }(z)-4\lambda_{11}\phi_{4}(z)]r^{3}\\ D_{z}=[2e_{31}u_{1}(z)+e_{33}w_{0}^{\prime }(z)-\lambda_{33}\phi_{0}^{\prime }(z)]+[4e_{31}u_{3}(z)+e_{33}w_{2}^{\prime }(z)-\lambda_{33}\phi_{2}^{\prime }(z)]r^{2}\\ \;+[e_{33}w_{4}^{\prime }(z)-\lambda_{33}\phi_{4}^{\prime }(z)]r^{4} \end{array}$$

Then, substituting Equation (10) into Equations (2) and (3), respectively, we can also obtain

(11) $$\begin{array}{l} \left\{[8c_{11}u_{3}(z)+2e_{31}{\phi^{\prime }}_{2}(z)+2c_{13}{w^{\prime }}_{2}(z)]+{\left[2c_{44}w_{2}(z)+c_{44}{u^{\prime }}_{1}(z)+2e_{15}\phi_{2}(z)\right]}_{,z}\right\}r\\ +\left\{[4c_{13}{w^{\prime }}_{4}(z)+4e_{31}{\phi^{\prime }}_{4}(z)]+{\left[4c_{44}w_{4}(z)+c_{44}{u^{\prime }}_{3}(z)+4e_{15}\phi_{4}(z)\right]}_{,z}\right\}r^{3}=0 \end{array},$$

(12) $$\begin{array}{l} \left\{[4c_{44}w_{2}(z)+2c_{44}{u^{\prime }}_{1}(z)+4e_{15}\phi_{2}(z)]+{\left[2c_{13}u_{1}(z)+c_{33}{w^{\prime }}_{0}(z)+e_{33}{\phi^{\prime }}_{0}(z)\right]}_{,z}\right\}\\ +\left\{[16c_{44}w_{4}(z)+4c_{44}{u^{\prime }}_{3}(z)+16e_{15}\phi_{4}(z)]+{\left[4c_{13}u_{3}(z)+c_{33}{w^{\prime }}_{2}(z)+e_{33}{\phi^{\prime }}_{2}(z)\right]}_{,z}\right\}r^{2}\\ +{\left[e_{33}\phi_{4}^{\prime }(z)+c_{33}w_{4}^{\prime }(z)\right]}_{,z}r^{4}=0 \end{array},$$

(13) $$\begin{array}{l} \left\{[4e_{15}w_{2}(z)+2e_{15}u_{1}^{\prime }(z)-4\lambda_{11}\phi_{2}(z)]+{\left[2e_{31}u_{1}(z)+e_{33}w_{0}^{\prime }(z)-\lambda_{33}\phi_{0}^{\prime }(z)\right]}_{,z}\right\}\\ +\left\{[16e_{15}w_{4}(z)+4e_{15}u_{3}^{\prime }(z)-16\lambda_{11}\phi_{4}(z)]+{\left[4e_{31}u_{3}(z)+e_{33}w_{2}^{\prime }(z)-\lambda_{33}\phi_{2}^{\prime }(z)\right]}_{,z}\right\}r^{2}\\ +{\left[e_{33}w_{4}^{\prime }(z)-\lambda_{33}\phi_{4}^{\prime }(z)\right]}_{,z}r^{4}=0 \end{array}.$$

From Equations (11)–(13), we can obtain

(14) $${\left[e_{33}\phi_{4}^{\prime }(z)+c_{33}w_{4}^{\prime }(z)\right]}_{,z}=0,$$

(15) $${\left[e_{33}w_{4}^{\prime }(z)-\lambda_{33}\phi_{4}^{\prime }(z)\right]}_{,z}=0,$$

(16) $$[4c_{13}w_{4}^{\prime }(z)+4e_{31}\phi_{4}^{\prime }(z)]+{\left[4c_{44}w_{4}(z)+c_{44}u_{3}^{\prime }(z)+4e_{15}\phi_{4}(z)\right]}_{,z}=0,$$

(17) $$[16c_{44}w_{4}(z)+4c_{44}u_{3}^{\prime }(z)+16e_{15}\phi_{4}(z)]+{\left[4c_{13}u_{3}(z)+c_{33}w_{2}^{\prime }(z)+e_{33}\phi_{2}^{\prime }(z)\right]}_{,z}=0,$$

(18) $$[16e_{15}w_{4}(z)+4e_{15}u_{3}^{\prime }(z)-16\lambda_{11}\phi_{4}(z)]+{\left[4e_{31}u_{3}(z)+e_{33}w_{2}^{\prime }(z)-\lambda_{33}\phi_{2}^{\prime }(z)\right]}_{,z}=0,$$

(19) $$[8c_{11}u_{3}(z)+2e_{31}\phi_{2}^{\prime }(z)+2c_{13}w_{2}^{\prime }(z)]+{\left[2c_{44}w_{2}(z)+c_{44}u_{1}^{\prime }(z)+2e_{15}\phi_{2}(z)\right]}_{,z}=0,$$

(20) $$[4c_{44}w_{2}(z)+2c_{44}u_{1}^{\prime }(z)+4e_{15}\phi_{2}(z)]+{\left[2c_{13}u_{1}(z)+c_{33}w_{0}^{\prime }(z)+e_{33}\phi_{0}^{\prime }(z)\right]}_{,z}=0,$$

(21) $$[4e_{15}w_{2}(z)+2e_{15}u_{1}^{\prime }(z)-4\lambda_{11}\phi_{2}(z)]+{\left[2e_{31}u_{1}(z)+e_{33}w_{0}^{\prime }(z)-\lambda_{33}\phi_{0}^{\prime }(z)\right]}_{,z}=0.$$

Substituting Equation (10) into Equation (7a,b), respectively, we can obtain

(22) $$\begin{array}{l} {}[c_{33}w_{4}^{\prime }(z)+e_{33}\phi_{4}^{\prime }(z)]{|}_{z=\pm h/2}=0\\ {}[e_{33}w_{4}^{\prime }(z)-\lambda_{33}\phi_{4}^{\prime }(z)]{|}_{z=\pm h/2}=0\\ {}[4c_{44}w_{4}(z)+c_{44}u_{3}^{\prime }(z)+4e_{15}\phi_{4}(z)]{|}_{z=\pm h/2}=0\\ {}[4c_{13}u_{3}(z)+c_{33}w_{2}^{\prime }(z)+e_{33}\phi_{2}^{\prime }(z)]{|}_{z=\pm h/2}=0\\ {}[4e_{31}u_{3}(z)+e_{33}w_{2}^{\prime }(z)-\lambda_{33}\phi_{2}^{\prime }(z)]{|}_{z=\pm h/2}=0\\ {}[2c_{44}w_{2}(z)+c_{44}u_{1}^{\prime }(z)+2e_{15}\phi_{2}(z)]{|}_{z=\pm h/2}=0\\ {}[2e_{31}u_{1}(z)+e_{33}w_{0}^{\prime }(z)-\lambda_{33}\phi_{0}^{\prime }(z)]{|}_{z=\pm h/2}=0\\ {}[2c_{13}u_{1}(z)+c_{33}w_{0}^{\prime }(z)+e_{33}\phi_{0}^{\prime }(z)]{|}_{z=-h/2}=-q\\ {}[2c_{13}u_{1}(z)+c_{33}w_{0}^{\prime }(z)+e_{33}\phi_{0}^{\prime }(z)]{|}_{z=h/2}=0 \end{array}.$$

We can obtain from the integration of Equations (14) and (15), respectively,

(23a) $$e_{33}\phi_{4}^{\prime }(z)+c_{33}w_{4}^{\prime }(z)=b_{0},$$

(23b) $$e_{33}w_{4}^{\prime }(z)-\lambda_{33}\phi_{4}^{\prime }(z)=b_{1}.$$

Substituting Equation (23a,b) into the first and second ones of Equation (22), we can obtain

(24) $$b_{0}=0,b_{1}=0.$$

From Equations (23a,b) and (24), we can obtain

(25a) $$(e_{33}^{2}+\lambda_{33}c_{33})w_{4}^{\prime }(z)=0,$$

(25b) $$(e_{33}^{2}+\lambda_{33}c_{33})\phi_{4}^{\prime }(z)=0.$$

As we all know, $(e_{33}^{2}+\lambda_{33}c_{33})\neq 0$, thus

(26a) $$w_{4}^{\prime }(z)=0,$$

(26b) $$\phi_{4}^{\prime }(z)=0.$$

We can obtain from the integration of Equation (26a,b), respectively,

(27a) $$w_{4}(z)=a_{0},$$

(27b) $$\phi_{4}(z)=a_{1},$$

in which, $a_{0}$, $a_{1}$ are integration constants. Substituting Equation (27a,b) into Equation (16), we can obtain

(28) $${\left[4c_{44}a_{0}+c_{44}u_{3}^{\prime }(z)+4e_{15}a_{1}\right]}_{,z}=0.$$

From the integration of Equation (28), one has

(29) $$4c_{44}a_{0}+c_{44}u_{3}^{\prime }(z)+4e_{15}a_{1}=b_{2}.$$

Then, substituting Equation (29) into the third one of Equation (22), we can obtain

(30) $$b_{2}=0.$$

Substituting Equation (30) into Equation (29) and integrating the two sides of Equation (29), it gives

(31) $$u_{3}(z)=-(4a_{0}+4\frac{e_{15}}{c_{44}}a_{1})z+a_{2},$$

in which $a_{2}$ is an integration constant. Substituting Equations (27a,b) and (31) into Equations (17) and (18), respectively, we can obtain

(32) $${\left[4c_{13}u_{3}(z)+c_{33}w_{2}^{\prime }(z)+e_{33}\phi_{2}^{\prime }(z)\right]}_{,z}=0,$$

(33) $${\left[4e_{31}u_{3}(z)+e_{33}w_{2}^{\prime }(z)-\lambda_{33}\phi_{2}^{\prime }(z)\right]}_{,z}=(16e_{15}\frac{e_{15}}{c_{44}}+16\lambda_{11})a_{1}.$$

Integrating the two sides of Equations (32) and (33), we can obtain

(34) $$4c_{13}u_{3}(z)+c_{33}w_{2}^{\prime }(z)+e_{33}\phi_{2}^{\prime }(z)=b_{3},$$

(35) $$4e_{31}u_{3}(z)+e_{33}w_{2}^{\prime }(z)-\lambda_{33}\phi_{2}^{\prime }(z)=(16\frac{{\left(e_{15}^{0}\right)}^{2}}{c_{44}^{0}}+16\lambda_{11}^{0})a_{1}\int_{-h/2}^{z}f(z)dz+b_{4}.$$

Then, substituting Equations (34) and (35) into the fourth and fifth ones of Equation (22), we can obtain

(36) $$b_{3}=0,\;b_{4}=0,\;a_{1}=0.$$

Substituting Equation (36) into Equations (34) and (35), respectively, we can obtain

(37) $$4c_{13}u_{3}(z)+c_{33}w_{2}^{\prime }(z)+e_{33}\phi_{2}^{\prime }(z)=0,$$

(38) $$4e_{31}u_{3}(z)+e_{33}w_{2}^{\prime }(z)-\lambda_{33}\phi_{2}^{\prime }(z)=0.$$

From Equations (31), (37), and (38), we have

(39) $$w_{2}^{\prime }(z)=\frac{\left(4\lambda_{33}c_{13}+4e_{33}e_{31}\right)}{\left(\lambda_{33}c_{33}+e_{33}^{2}\right)}(4a_{0}z-a_{2}),$$

(40) $$\phi_{2}^{\prime }(z)=\frac{\left(4e_{33}c_{13}-4c_{33}e_{31}\right)}{\left(\lambda_{33}c_{33}+e_{33}^{2}\right)}(4a_{0}z-a_{2}).$$

Integrating the two sides of Equations (32) and (33), we can obtain

(41) $$w_{2}(z)=\frac{\left(4\lambda_{33}c_{13}+4e_{33}e_{31}\right)}{\left(\lambda_{33}c_{33}+e_{33}^{2}\right)}(2a_{0}z^{2}-a_{2}z)+a_{3},$$

(42) $$\phi_{2}(z)=\frac{\left(4e_{33}c_{13}-4c_{33}e_{31}\right)}{\left(\lambda_{33}c_{33}+e_{33}^{2}\right)}(2a_{0}z^{2}-a_{2}z)+a_{4},$$

in which $a_{3}$, $a_{4}$ are integration constants. From Equations (31), (41), and (42), Equation (19) gives

(43) $$\begin{array}{l} {\left[2c_{44}w_{2}(z)+c_{44}u_{1}^{\prime }(z)+2e_{15}\phi_{2}(z)\right]}_{,z}=(8c_{11}\lambda_{33}c_{33}+8c_{11}e_{33}^{2}+8c_{33}e_{31}^{2}\\ -8e_{31}e_{33}c_{13}-8\lambda_{33}c_{13}^{2}-8c_{13}e_{33}e_{31})\frac{\left(4a_{0}z-a_{2}\right)}{\lambda_{33}c_{33}+e_{33}^{2}} \end{array}.$$

Integrating the two sides of Equation (43), we can obtain

(44) $$[2c_{44}w_{2}(z)+c_{44}u_{1}^{\prime }(z)+2e_{15}\phi_{2}(z)]=4a_{0}K_{0}F_{1}(z)-a_{2}K_{0}F_{0}(z)+b_{5},$$

in which $K_{0}=8\frac{\left(c_{11}^{0}\lambda_{33}^{0}c_{33}^{0}+c_{11}^{0}e_{33}^{0}e_{33}^{0}+c_{33}^{0}e_{31}^{0}e_{31}^{0}-e_{31}^{0}e_{33}^{0}c_{13}^{0}-\lambda_{33}^{0}c_{13}^{0}c_{13}^{0}-c_{13}^{0}e_{33}^{0}e_{31}^{0}\right)}{\lambda_{33}^{0}c_{33}^{0}+e_{33}^{0}e_{33}^{0}}$, $F_{0}(z)=\int_{-h/2}^{z}f(z)dz$, $F_{1}(z)=\int_{-h/2}^{z}zf(z)dz$. Substituting Equation (44) into the sixth one of Equation (22), we can obtain

(45) $$b_{5}=0,\;4a_{0}F_{1}(h/2)-a_{2}F_{0}(h/2)=0.$$

From the second one of Equation (45), we can obtain

(46) $$a_{2}=4\frac{F_{1}(h/2)}{F_{0}(h/2)}a_{0}.$$

Substituting Equations (41) and (42) into Equation (44) and with the help of Equations (45) and (46), we get

(47) $$u_{1}^{\prime }(z)=4a_{0}\frac{K_{0}}{c_{44}^{0}}\frac{F_{1}(z)}{f(z)}-a_{2}\frac{K_{0}}{c_{44}^{0}}\frac{F_{0}(z)}{f(z)}-K_{1}(2a_{0}z^{2}-a_{2}z)-2a_{3}-2\frac{e_{15}}{c_{44}}a_{4},$$

in which $K_{1}=\frac{\left(8c_{44}\lambda_{33}c_{13}+8c_{44}e_{33}e_{31}+8e_{15}e_{33}c_{13}-8e_{15}c_{33}e_{31}\right)}{c_{44}(\lambda_{33}c_{33}+e_{33}^{2})}$. Integrating the two sides of Equation (47), one has

(48) $$u_{1}(z)=4a_{0}\frac{K_{0}}{c_{44}^{0}}H_{1}(z)-a_{2}\frac{K_{0}}{c_{44}^{0}}H_{0}(z)-\frac{2}{3}K_{1}a_{0}z^{3}+K_{1}a_{2}\frac{z^{2}}{2}-(2a_{3}+2\frac{e_{15}}{c_{44}}a_{4})z+a_{5},$$

in which $H_{0}(z)=\int_{-h/2}^{z}\frac{F_{0}(z)}{f(z)}dz,\;H_{1}(z)=\int_{-h/2}^{z}\frac{F_{1}(z)}{f(z)}dz$. Substituting Equations (41), (42), and (48) into Equations (20) and (21), respectively, we can obtain

(49) $${\left[2c_{13}u_{1}(z)+c_{33}w_{0}^{\prime }(z)+e_{33}\phi_{0}^{\prime }(z)\right]}_{,z}=2a_{2}K_{0}F_{0}(z)-8a_{0}K_{0}F_{1}(z),$$

(50) $$\begin{array}{l} {\left[2e_{31}u_{1}(z)+e_{33}w_{0}^{\prime }(z)-\lambda_{33}\phi_{0}^{\prime }(z)\right]}_{,z}=2a_{2}K_{0}\frac{e_{15}}{c_{44}}F_{0}(z)-8a_{0}K_{0}\frac{e_{15}}{c_{44}}F_{1}(z)\\ -(4e_{15}\frac{e_{15}}{c_{44}}+4\lambda_{11})(8K_{2}a_{0}z^{2}-4K_{2}a_{2}z-a_{4}) \end{array},$$

in which $K_{2}=\frac{c_{33}e_{31}-e_{33}c_{13}}{\lambda_{33}c_{33}+e_{33}^{2}}$. Integrating the two sides of Equations (49) and (50), respectively, we get

(51) $$[2c_{13}u_{1}(z)+c_{33}w_{0}^{\prime }(z)+e_{33}\phi_{0}^{\prime }(z)]=2a_{2}K_{0}G_{0}(z)-8a_{0}K_{0}G_{1}(z)+b_{6},$$

(52) $$\begin{array}{l} \left[2e_{31}u_{1}(z)+e_{33}w_{0}^{\prime }(z)-\lambda_{33}\phi_{0}^{\prime }(z)]=2a_{2}K_{0}\frac{e_{15}}{c_{44}}G_{0}(z)-8a_{0}K_{0}\frac{e_{15}}{c_{44}}G_{1}(z\right)\\ -(4e_{15}^{0}\frac{e_{15}}{c_{44}}+4\lambda_{11}^{0})[8K_{2}a_{0}F_{2}(z)-4K_{2}a_{2}F_{1}(z)-a_{4}F_{0}(z)]+b_{7} \end{array},$$

in which $G_{0}(z)=\int_{-h/2}^{z}F_{0}(z)dz,\;G_{1}(z)=\int_{-h/2}^{z}F_{1}(z)dz,\;F_{2}(z)=\int_{-h/2}^{z}z^{2}f(z)dz$. Substituting Equations (51) and (52) into the seventh, eighth, and ninth ones of Equation (22), respectively, we obtain the following

(53) $$b_{7}=0,$$

(54) $$a_{4}=K_{3}a_{0},$$

(55) $$b_{6}=-q,$$

(56) $$a_{0}=K_{4}q,$$

in which

$$K_{3}=8K_{2}\frac{F_{2}(h/2)}{F_{0}(h/2)}-16K_{2}\frac{F_{1}^{2}(h/2)}{F_{0}^{2}(h/2)}-2\frac{e_{15}^{0}K_{0}G_{0}(h/2)F_{1}(h/2)}{\left(e_{15}^{0}e_{15}^{0}+c_{44}^{0}\lambda_{11}^{0})F_{0}^{2}(h/2\right)}+2\frac{e_{15}^{0}K_{0}G_{1}(h/2)}{\left(e_{15}^{0}e_{15}^{0}+c_{44}^{0}\lambda_{11}^{0})F_{0}(h/2\right)}$$

$$K_{4}=\frac{F_{0}(h/2)}{\left[8F_{1}(h/2)K_{0}G_{0}(h/2)-8F_{0}(h/2)K_{0}G_{1}(h/2)\right]}$$

Substituting Equation (48) into Equations (51) and (52), respectively, we get

(57) $$\begin{array}{l} c_{33}w_{0}^{\prime }(z)+e_{33}\phi_{0}^{\prime }(z)=2a_{2}K_{0}G_{0}(z)-8a_{0}K_{0}G_{1}(z)-q-8c_{13}a_{0}\frac{K_{0}}{c_{44}^{0}}H_{1}(z)\\ +2c_{13}a_{2}\frac{K_{0}}{c_{44}^{0}}H_{0}(z)+\frac{4}{3}c_{13}K_{1}a_{0}z^{3}-c_{13}K_{1}a_{2}z^{2}+(4a_{3}c_{13}+4c_{13}\frac{e_{15}}{c_{44}}a_{4})z-2c_{13}a_{5} \end{array},$$

(58) $$\begin{array}{l} e_{33}w_{0}^{\prime }(z)-\lambda_{33}\phi_{0}^{\prime }(z)=-(4e_{15}^{0}\frac{e_{15}}{c_{44}}+4\lambda_{11}^{0})[8K_{2}a_{0}F_{2}(z)-4K_{2}a_{2}F_{1}(z)-a_{4}F_{0}(z)]\\ +2a_{2}K_{0}\frac{e_{15}}{c_{44}}G_{0}(z)-8a_{0}K_{0}\frac{e_{15}}{c_{44}}G_{1}(z)-8e_{31}a_{0}\frac{K_{0}}{c_{44}^{0}}H_{1}(z)+2e_{31}a_{2}\frac{K_{0}}{c_{44}^{0}}H_{0}(z)\\ +\frac{4}{3}e_{31}K_{1}a_{0}z^{3}-e_{31}K_{1}a_{2}z^{2}+(4a_{3}e_{31}+4e_{31}\frac{e_{15}}{c_{44}}a_{4})z-2e_{31}a_{5} \end{array},$$

From Equations (57) and (58), we can obtain

(59) $$w_{0}^{\prime }(z)=J_{0}(z)a_{0}+J_{1}(z)a_{2}+J_{2}(z)a_{3}+J_{3}(z)a_{4}+J_{4}(z)a_{5}+J_{5}(z)q,$$

(60) $$\phi_{0}^{\prime }(z)=L_{0}(z)a_{0}+L_{1}(z)a_{2}+L_{2}(z)a_{3}+L_{3}(z)a_{4}+L_{4}(z)a_{5}+L_{5}(z)q,$$

in which

$$\begin{array}{l} J_{0}(z)=\frac{1}{\left(\lambda_{33}^{0}c_{33}^{0}+e_{33}^{0}e_{33}^{0}\right)}[-8K_{0}\frac{G_{1}(z)}{f(z)}(\lambda_{33}^{0}+e_{33}^{0}\frac{e_{15}}{c_{44}})-8(\lambda_{33}^{0}c_{13}^{0}+e_{33}^{0}e_{31}^{0})\frac{K_{0}}{c_{44}^{0}}H_{1}(z)\\ +\frac{4}{3}(\lambda_{33}^{0}c_{13}^{0}+e_{33}^{0}e_{31}^{0})K_{1}z^{3}-32e_{33}^{0}(e_{15}^{0}\frac{e_{15}}{c_{44}}+\lambda_{11}^{0})K_{2}\frac{F_{2}(z)}{f(z)}] \end{array},$$

$$\begin{array}{l} J_{1}(z)=\frac{1}{\left(\lambda_{33}^{0}c_{33}^{0}+e_{33}^{0}e_{33}^{0}\right)}[2(\lambda_{33}^{0}+e_{33}^{0}\frac{e_{15}}{c_{44}})K_{0}\frac{G_{0}(z)}{f(z)}+2(\lambda_{33}^{0}c_{13}^{0}+e_{33}^{0}e_{31}^{0})\frac{K_{0}}{c_{44}^{0}}H_{0}(z)\\ -(\lambda_{33}^{0}c_{13}^{0}+e_{33}^{0}e_{31}^{0})K_{1}z^{2}+16e_{33}^{0}(e_{15}^{0}\frac{e_{15}}{c_{44}}+\lambda_{11}^{0})K_{2}\frac{F_{1}(z)}{f(z)}] \end{array},$$

$$J_{2}(z)=4\frac{\left(\lambda_{33}^{0}c_{13}^{0}+e_{33}^{0}e_{31}^{0}\right)}{\left(\lambda_{33}^{0}c_{33}^{0}+e_{33}^{0}e_{33}^{0}\right)}z,$$

$$J_{3}(z)=\frac{1}{\left(\lambda_{33}^{0}c_{33}^{0}+e_{33}^{0}e_{33}^{0}\right)}[4(\lambda_{33}^{0}c_{13}^{0}+e_{33}^{0}e_{31}^{0})\frac{e_{15}}{c_{44}}z+4e_{33}^{0}(e_{15}^{0}\frac{e_{15}}{c_{44}}+\lambda_{11}^{0})\frac{F_{0}(z)}{f(z)}],$$

$$J_{4}(z)=-2\frac{\left(\lambda_{33}^{0}c_{13}^{0}+e_{33}^{0}e_{31}^{0}\right)}{\left(\lambda_{33}^{0}c_{33}^{0}+e_{33}^{0}e_{33}^{0}\right)},$$

$$J_{5}(z)=-\frac{\lambda_{33}^{0}}{\left(\lambda_{33}^{0}c_{33}^{0}+e_{33}^{0}e_{33}^{0}\right)}\frac{1}{f(z)},$$

$$\begin{array}{l} L_{0}(z)=\frac{1}{\left(e_{33}^{0}e_{33}^{0}+\lambda_{33}^{0}c_{33}^{0}\right)}[8(c_{33}^{0}\frac{e_{15}}{c_{44}}-e_{33}^{0})K_{0}\frac{G_{1}(z)}{f(z)}+8(c_{33}^{0}e_{31}^{0}-e_{33}^{0}c_{13}^{0})\frac{K_{0}}{c_{44}^{0}}H_{1}(z)\\ +\frac{4}{3}(e_{33}^{0}c_{13}^{0}-c_{33}^{0}e_{31}^{0})K_{1}z^{3}+32c_{33}^{0}(e_{15}^{0}\frac{e_{15}}{c_{44}}+\lambda_{11}^{0})K_{2}\frac{F_{2}(z)}{f(z)}] \end{array},$$

$$\begin{array}{l} L_{1}(z)=\frac{1}{\left(e_{33}^{0}e_{33}^{0}+\lambda_{33}^{0}c_{33}^{0}\right)}[2(e_{33}^{0}-c_{33}^{0}\frac{e_{15}}{c_{44}})K_{0}\frac{G_{0}(z)}{f(z)}+2(e_{33}^{0}c_{13}^{0}-c_{33}^{0}e_{31}^{0})\frac{K_{0}}{c_{44}^{0}}H_{0}(z)\\ +(c_{33}^{0}e_{31}^{0}-e_{33}^{0}c_{13}^{0})K_{1}z^{2}-16c_{33}^{0}(e_{15}^{0}\frac{e_{15}}{c_{44}}+\lambda_{11}^{0})K_{2}\frac{F_{1}(z)}{f(z)}] \end{array},$$

$$L_{2}(z)=4\frac{\left(e_{33}^{0}c_{13}^{0}-c_{33}^{0}e_{31}^{0}\right)}{\left(e_{33}^{0}e_{33}^{0}+\lambda_{33}^{0}c_{33}^{0}\right)}z,$$

$$L_{3}(z)=\frac{1}{\left(e_{33}^{0}e_{33}^{0}+\lambda_{33}^{0}c_{33}^{0}\right)}[4(e_{33}^{0}c_{13}^{0}-c_{33}^{0}e_{31}^{0})\frac{e_{15}}{c_{44}}z-4c_{33}^{0}(e_{15}^{0}\frac{e_{15}}{c_{44}}+\lambda_{11}^{0})\frac{F_{0}(z)}{f(z)}],$$

$$L_{4}(z)=2\frac{\left(c_{33}^{0}e_{31}^{0}-e_{33}^{0}c_{13}^{0}\right)}{\left(e_{33}^{0}e_{33}^{0}+\lambda_{33}^{0}c_{33}^{0}\right)},$$

$$L_{5}(z)=-\frac{e_{33}^{0}}{\left(e_{33}^{0}e_{33}^{0}+\lambda_{33}^{0}c_{33}^{0}\right)}\frac{1}{f(z)}.$$

Integrating the two sides of Equations (59) and (60), respectively, we can obtain

(61) $$w_{0}(z)=j_{0}(z)a_{0}+j_{1}(z)a_{2}+j_{2}(z)a_{3}+j_{3}(z)a_{4}+j_{4}(z)a_{5}+j_{5}(z)q+a_{6},$$

(62) $$\phi_{0}(z)=l_{0}(z)a_{0}+l_{1}(z)a_{2}+l_{2}(z)a_{3}+l_{3}(z)a_{4}+l_{4}(z)a_{5}+l_{5}(z)q+a_{7},$$

in which $j_{i}(z)=\int_{-h/2}^{z}J_{i}(z)dz,\;l_{i}(z)=\int_{-h/2}^{z}L_{i}(z)dz,\;(i=0,1,\ldots ,5)$.

From the above process, it can be seen that there are 8 integration constants $a_{i}(i=0,1,\ldots ,7)$ in total, in which $a_{0},a_{1},a_{2},a_{4}$ have been determined and $a_{3},a_{5},a_{6},a_{7}$ can be determined by the boundary conditions at r=a.

Substituting the displacement functions $u_{i}(z)$, $w_{i}(z)$, and the electric potential function ϕ(z) into Equation (10), the expressions of elastic stress and electric displacement components of the circular plate can be obtained

(63) $$\begin{array}{l} \sigma_{r}=(c_{11}+c_{12})[4a_{0}\frac{K_{0}}{c_{44}^{0}}H_{1}(z)-a_{2}\frac{K_{0}}{c_{44}^{0}}H_{0}(z)-\frac{2}{3}K_{1}a_{0}z^{3}+K_{1}a_{2}\frac{z^{2}}{2}-(2a_{3}+2\frac{e_{15}}{c_{44}}a_{4})z+a_{5}]\\ +K_{5}f(z)(4a_{0}z-a_{2})r^{2}+c_{13}[J_{0}(z)a_{0}+J_{1}(z)a_{2}+J_{2}(z)a_{3}+J_{3}(z)a_{4}+J_{4}(z)a_{5}+J_{5}(z)q]\\ +e_{31}[L_{0}(z)a_{0}+L_{1}(z)a_{2}+L_{2}(z)a_{3}+L_{3}(z)a_{4}+L_{4}(z)a_{5}+L_{5}(z)q] \end{array},$$

(64) $$\begin{array}{l} \sigma_{\theta }=(c_{12}+c_{11})[4a_{0}\frac{K_{0}}{c_{44}^{0}}H_{1}(z)-a_{2}\frac{K_{0}}{c_{44}^{0}}H_{0}(z)-\frac{2}{3}K_{1}a_{0}z^{3}+K_{1}a_{2}\frac{z^{2}}{2}-(2a_{3}+2\frac{e_{15}}{c_{44}}a_{4})z+a_{5}]\\ +K_{6}f(z)(4a_{0}z-a_{2})r^{2}+c_{13}[J_{0}(z)a_{0}+J_{1}(z)a_{2}+J_{2}(z)a_{3}+J_{3}(z)a_{4}+J_{4}(z)a_{5}+J_{5}(z)q]\\ +e_{31}[L_{0}(z)a_{0}+L_{1}(z)a_{2}+L_{2}(z)a_{3}+L_{3}(z)a_{4}+L_{4}(z)a_{5}+L_{5}(z)q] \end{array},$$

(65) $$\begin{array}{l} \sigma_{z}=2c_{13}[4a_{0}\frac{K_{0}}{c_{44}^{0}}H_{1}(z)-a_{2}\frac{K_{0}}{c_{44}^{0}}H_{0}(z)-\frac{2}{3}K_{1}a_{0}z^{3}+K_{1}a_{2}\frac{z^{2}}{2}-(2a_{3}+2\frac{e_{15}}{c_{44}}a_{4})z+a_{5}]\\ +c_{33}[J_{0}(z)a_{0}+J_{1}(z)a_{2}+J_{2}(z)a_{3}+J_{3}(z)a_{4}+J_{4}(z)a_{5}+J_{5}(z)q]\\ +e_{33}[L_{0}(z)a_{0}+L_{1}(z)a_{2}+L_{2}(z)a_{3}+L_{3}(z)a_{4}+L_{4}(z)a_{5}+L_{5}(z)q] \end{array},$$

(66) $$\tau_{zr}=[4a_{0}K_{0}F_{1}(z)-a_{2}K_{0}F_{0}(z)]r,$$

(67) $$\begin{array}{l} D_{r}=[8\frac{\left(c_{44}\lambda_{11}c_{33}e_{31}-c_{44}\lambda_{11}e_{33}c_{13}+e_{15}e_{15}c_{33}e_{31}-e_{15}e_{15}e_{33}c_{13}\right)}{c_{44}(\lambda_{33}c_{33}+e_{33}^{2})}(2a_{0}z^{2}-a_{2}z)\\ +4e_{15}a_{0}\frac{K_{0}}{c_{44}^{0}}\frac{F_{1}(z)}{f(z)}-e_{15}a_{2}\frac{K_{0}}{c_{44}^{0}}\frac{F_{0}(z)}{f(z)}-2(\lambda_{11}+e_{15}\frac{e_{15}}{c_{44}})a_{4}]r \end{array},$$

(68) $$\begin{array}{l} D_{z}=8e_{31}a_{0}\frac{K_{0}}{c_{44}^{0}}H_{1}(z)-2e_{31}a_{2}\frac{K_{0}}{c_{44}^{0}}H_{0}(z)-\frac{4}{3}e_{31}K_{1}a_{0}z^{3}+e_{31}K_{1}a_{2}z^{2}-4e_{31}a_{3}z\\ -4e_{31}\frac{e_{15}}{c_{44}}a_{4}z+2e_{31}a_{5}+e_{33}[J_{0}(z)a_{0}+J_{1}(z)a_{2}+J_{2}(z)a_{3}+J_{3}(z)a_{4}+J_{4}(z)a_{5}\\ +J_{5}(z)q]-\lambda_{33}[L_{0}(z)a_{0}+L_{1}(z)a_{2}+L_{2}(z)a_{3}+L_{3}(z)a_{4}+L_{4}(z)a_{5}+L_{5}(z)q] \end{array},$$

in which

$$K_{5}=\frac{\left(4c_{13}\lambda_{33}c_{13}+4c_{13}e_{33}e_{31}+4e_{31}e_{33}c_{13}-4e_{31}c_{33}e_{31}-3c_{11}\lambda_{33}c_{33}-3c_{11}e_{33}^{2}-c_{12}\lambda_{33}c_{33}-c_{12}e_{33}^{2}\right)}{\left(\lambda_{33}c_{33}+e_{33}^{2}\right)},$$

$$K_{6}=\frac{\left(4c_{13}\lambda_{33}c_{13}+4c_{13}e_{33}e_{31}+4e_{31}e_{33}c_{13}-4e_{31}c_{33}e_{31}\right)}{\left(\lambda_{33}c_{33}+e_{33}^{2}\right)}-(3c_{12}+c_{11}).$$

The expressions of the radial force and bending moment are

(69) $$N(r)=\int_{-h/2}^{h/2}\sigma_{r}dz,$$

(70) $$M(r)=\int_{-h/2}^{h/2}z\sigma_{r}dz,$$

and the expressions of the elastic displacement and electric potential are

(71) $$\begin{array}{l} u_{r}(r,z)=[4a_{0}\frac{K_{0}}{c_{44}^{0}}H_{1}(z)-a_{2}\frac{K_{0}}{c_{44}^{0}}H_{0}(z)-\frac{2}{3}K_{1}a_{0}z^{3}+K_{1}a_{2}\frac{z^{2}}{2}\\ -(2a_{3}+2\frac{e_{15}}{c_{44}}a_{4})z+a_{5}]r+(a_{2}-4a_{0}z)r^{3} \end{array},$$

(72) $$\begin{array}{l} u_{z}(r,z)=j_{0}(z)a_{0}+j_{1}(z)a_{2}+j_{2}(z)a_{3}+j_{3}(z)a_{4}+j_{4}(z)a_{5}+j_{5}(z)q+a_{6}\\ +[\frac{\left(4\lambda_{33}c_{13}+4e_{33}e_{31}\right)}{\left(\lambda_{33}c_{33}+e_{33}^{2}\right)}(2a_{0}z^{2}-a_{2}z)+a_{3}]r^{2}+a_{0}r^{4} \end{array},$$

(73) $$\begin{array}{l} \phi (r,z)=l_{0}(z)a_{0}+l_{1}(z)a_{2}+l_{2}(z)a_{3}+l_{3}(z)a_{4}+l_{4}(z)a_{5}+l_{5}(z)q+a_{7}\\ +[\frac{\left(4e_{33}c_{13}-4c_{33}e_{31}\right)}{\left(\lambda_{33}c_{33}+e_{33}^{2}\right)}(2a_{0}z^{2}-a_{2}z)+a_{4}]r^{2} \end{array}.$$

From Equation (7c), we can obtain

(74) $$N(a)=\int_{-h/2}^{h/2}\sigma_{r}dz=\bar{N},$$

(75) $$M(a)=\int_{-h/2}^{h/2}z\sigma_{r}dz=\bar{M}.$$

There contain only two undetermined constants, $a_{3}$ and $a_{5}$, thus, from Equations (74) and (75), $a_{3}$ and $a_{5}$ can be determined. Then, from Equation (7c), one has

(76) $$u_{z}(a,0)=0.$$

With the help of determined $a_{3}$ and $a_{5}$, the undetermined constants $a_{6}$ can also be determined by Equation (76). Thus, we obtain the electroelastic solution of the axisymmetric deformation problem of simply supported functionally graded piezoelectric circular plates under the action of combined mechanical loads.

## 3. Comparisons and Discussions

### 3.1. Comparisions with Existing Result

**Figure 2:**
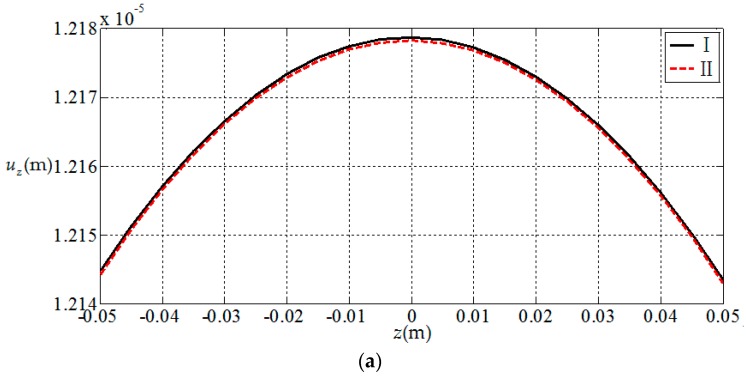

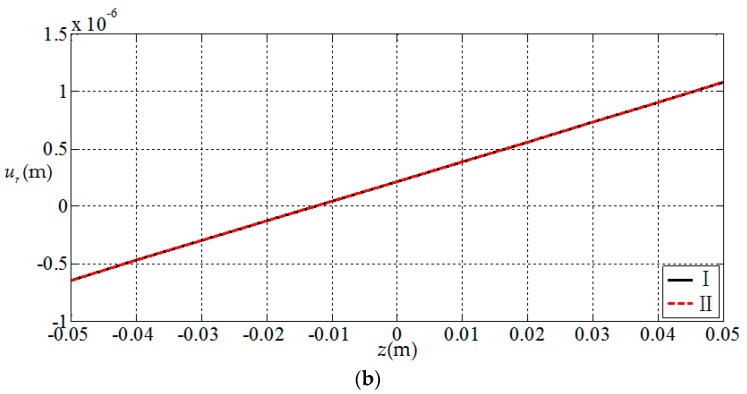
Variation of elastic displacements with coordinates z, where I denotes the solution presented in this study; II denotes the solution presented in [32]. (**a**) *z*-direction displacement at the center of plate $u_{z}(0,z)$; (**b**) radial displacement at the periphery of plate $u_{r}(1,z)$.

**Figure 3:**
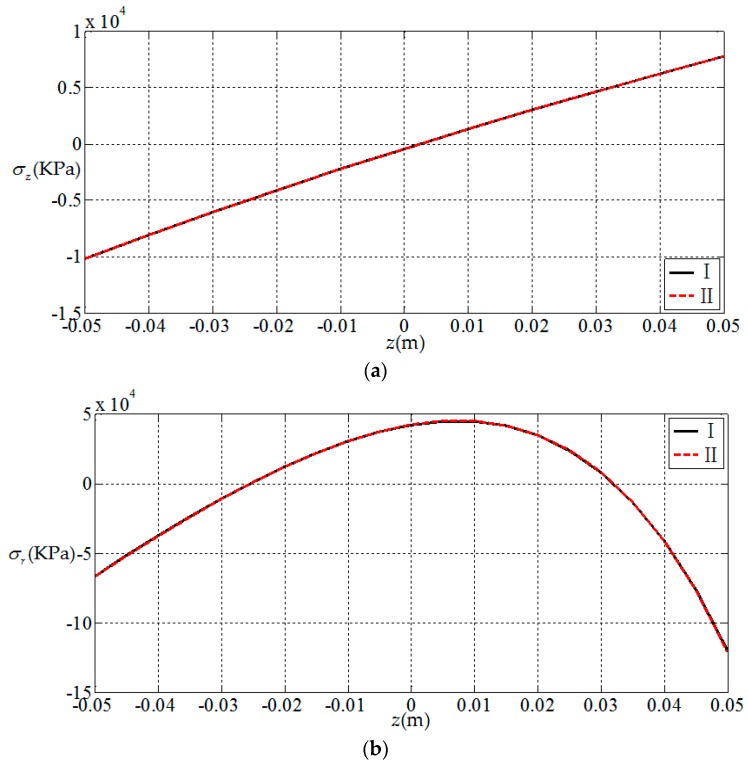
Variation of elastic stress with coordinates z, where I denotes the solution presented in this study; II denotes the solution presented in [32]. (**a**) *z*-direction stress at the periphery of plate $\sigma_{z}(1,z)$; (**b**) radial stress at the periphery of plate $\sigma_{r}(1,z)$.

**Figure 4:**
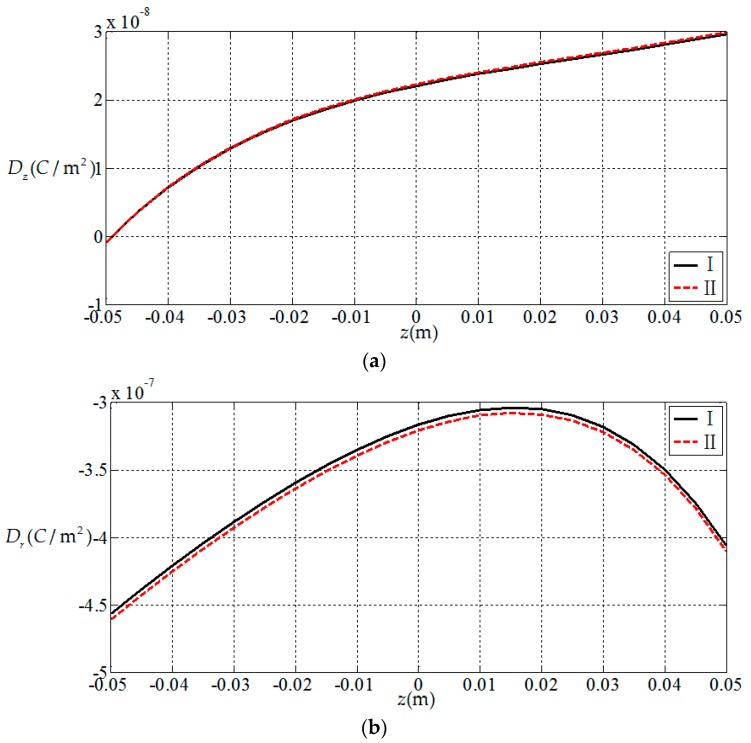
Variation of electric displacement with coordinates z, where I denotes the solution presented in this study; II denotes the solution presented in [32]. (**a**) Electric displacement at the periphery of plate $D_{z}(1,z)$; (**b**) electric displacement at the periphery of plate $D_{r}(1,z)$.

**Figure 5:**
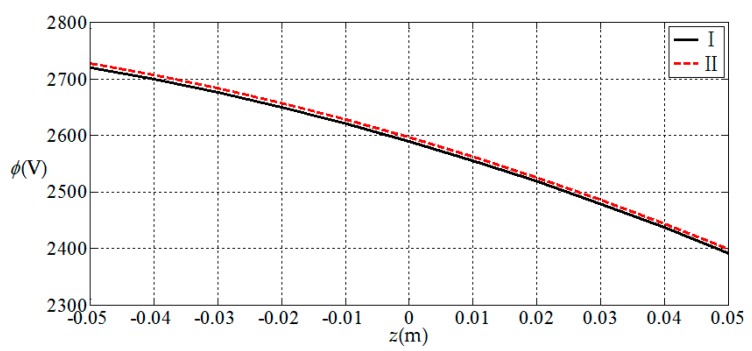
Variation of electric potential at the periphery of plate ϕ(1,z), where I denotes the solution presented in this study; II denotes the solution presented in [32].

Here, we use a numerical example to verify the results presented in this paper. Since there is no electroelastic solution for functionally graded piezoelectric circular plates under the action of combined mechanical loads, only the solution under a single load [32] is available, and we verify the correctness of the results presented in this paper according to the regression. That is, let the radial force and bending moment in this study be zero; the circular plate is now subjected to uniformly distributed loads only, thus the obtained result may be compared with the solution presented in [32] (subjected to uniformly distributed loads only). For this purpose, we consider a simply supported functionally graded piezoelectric circular plate with a=1 m, h=0.1 m and subjected to the action of uniformly-distributed loads q=1 KPa on the upper surface of the plate, in which $\bar{N}=0$ and $\bar{M}=0$ at the periphery of the plate. We here use two solutions, the solution presented in this study (denoted by I) and the solution presented in [32] (denoted by II), to conduct the numerical comparisons. In the comparisons, the functional gradient parameter α takes 2 and the material constants at z=0 are listed in Table 1. The comparison results are shown in Figure 2, Figure 3, Figure 4 and Figure 5, in which Figure 2 and Figure 3 show the elastic displacement and stress, respectively; Figure 4 and Figure 5 show the electric displacement and the electric potential, respectively. From Figure 2, Figure 3, Figure 4 and Figure 5, it can be found that the solution presented in this study (I) and the solution presented in the previous study (II) are very close to each other, which demonstrates the validity of the results presented in this study.

### 3.2. Influences of Functionally Graded Parameters

Let us consider another numerical example of a simply supported functionally graded piezoelectric circular plate with a=1 m, h=0.1 m and subjected to the action of uniformly distributed loads q=1 KPa on the upper surface of the plate and the action of the radial force $\bar{N}=6$ kN/m and the bending moment $\bar{M}=6$ kN at the periphery of the plate, to investigate the influence of different functionally graded parameters on the elastic displacement and elastic stress, as well as the electric displacement and electric potential of the circular plate. Suppose the functional gradient parameter α takes 0, 1, and 2, respectively. Besides, in the computation we still adopt the material constants at z=0 in Table 1.

**Figure 6:**
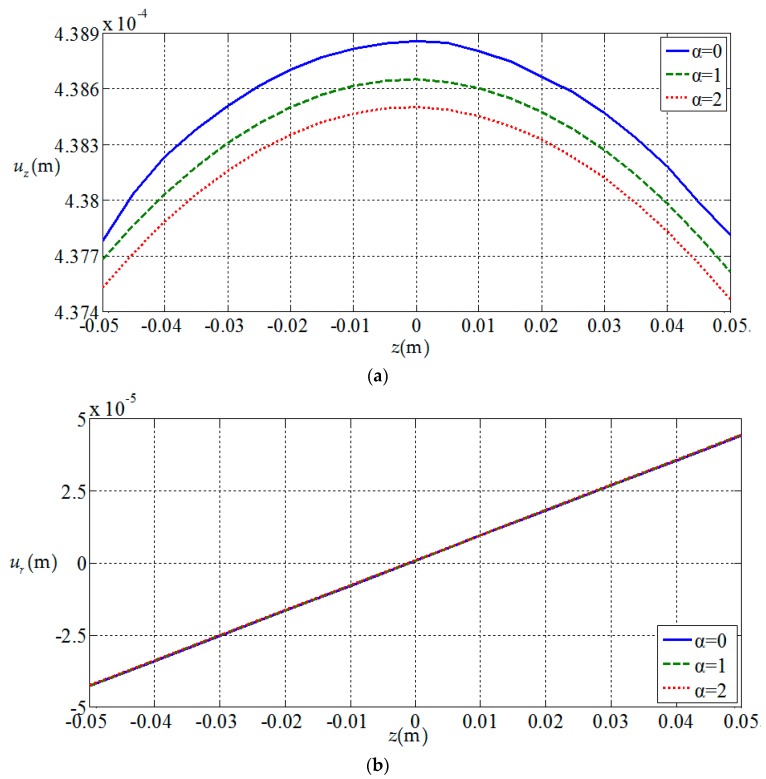
Variation of elastic displacements with coordinates z. (**a**) *z*-direction displacement at the center of plate $u_{z}(0,z)$; (**b**) radial displacement at the periphery of plate $u_{r}(1,z)$.

**Figure 7:**
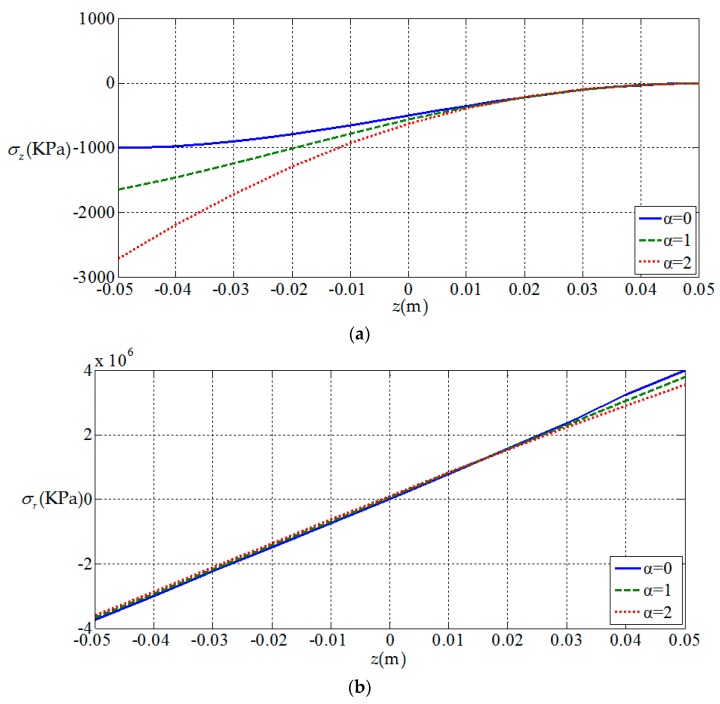
Variation of elastic stress with coordinates z. (**a**) *z*-direction stress at the periphery of plate $\sigma_{z}(1,z)$; (**b**) radial stress at the periphery of plate $\sigma_{r}(1,z)$.

**Figure 8:**
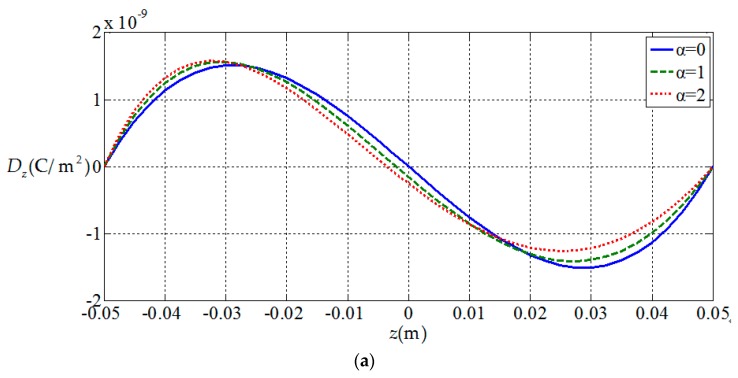

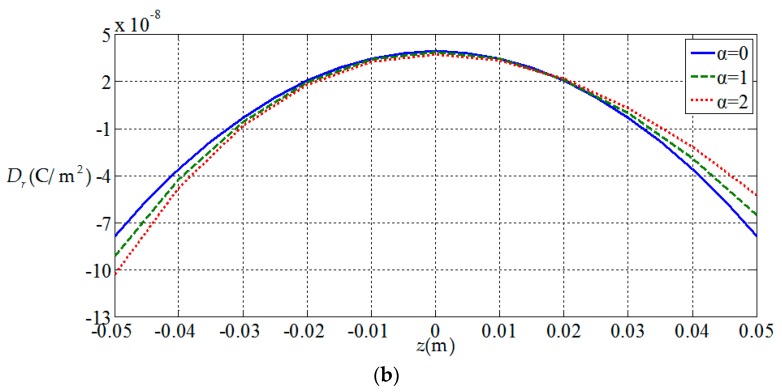
Variation of electric displacement with coordinates z. (**a**) Electric displacement at the periphery of plate $D_{z}(1,z)$; (**b**) electric displacement at the periphery of plate $D_{r}(1,z)$.

**Figure 9:**
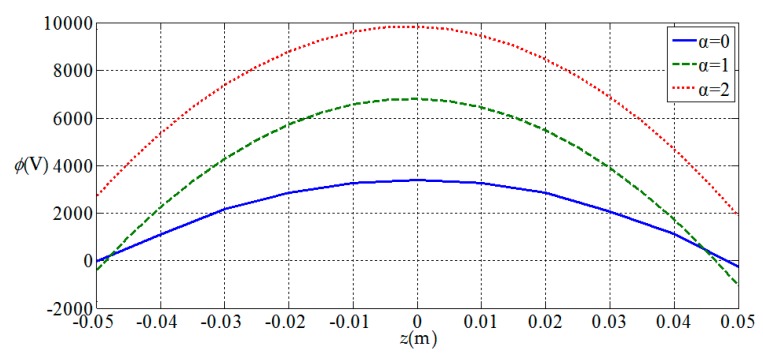
Variation of electric potential at the periphery of plate ϕ(1,z) with coordinates z.

Figure 6, Figure 7, Figure 8 and Figure 9, show the variation of the elastic displacement and stress, as well as the electric displacement and electric potential with the coordinate z. From Figure 6, Figure 7, Figure 8 and Figure 9 it can be found that the variation curves of all physical quantities of the functionally graded piezoelectric circular plate (α≠0) are deviated from the uniform piezoelectric plate (α=0), and the degree of deviation increases with the increase of functional gradient parameter α, in which the change of $u_{z}$, $\sigma_{z}$, $D_{r}$, $D_{z}$, ϕ are obvious, the change of $\sigma_{r}$ is relatively small, and $u_{r}$ has almost no change. For the functionally graded piezoelectric circular plate, $u_{r}$ and $\sigma_{r}$ change linearly along the thickness direction and $u_{z}$, $\sigma_{z}$, $D_{r}$, $D_{z}$ and ϕ change nonlinearly along the thickness direction.

**Figure 10:**
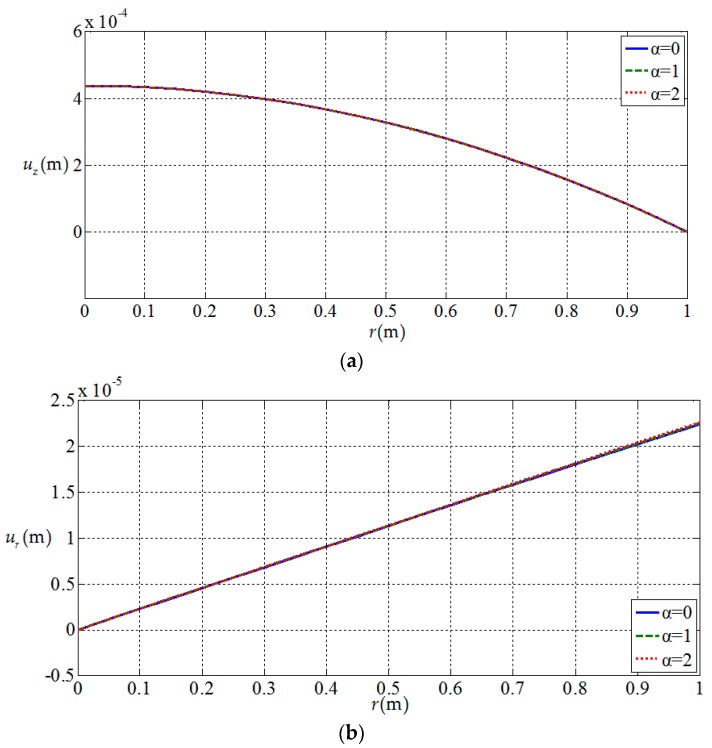
Variation of elastic displacement with coordinates r at z=h/4. (**a**) *z*-direction displacement $u_{z}(r,h/4)$ at z=h/4; (**b**) radial displacement $u_{r}(r,h/4)$ at z=h/4.

**Figure 11:**
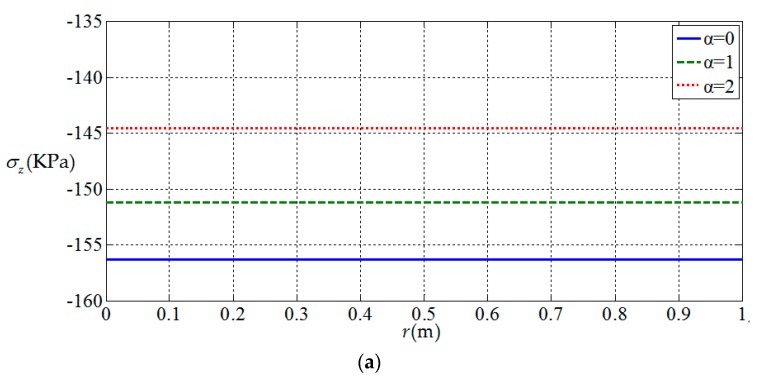

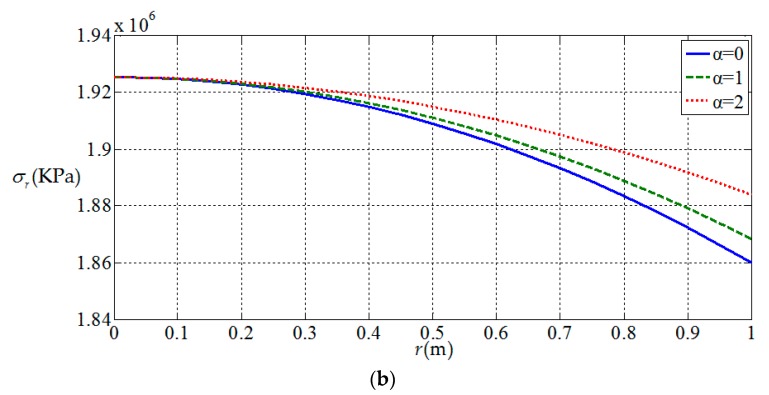
Variation of elastic stress with coordinates r at z=h/4. (**a**) *z*-direction stress $\sigma_{z}(r,h/4)$ at z=h/4; (**b**) radial stress $\sigma_{r}(r,h/4)$ at z=h/4.

**Figure 12:**
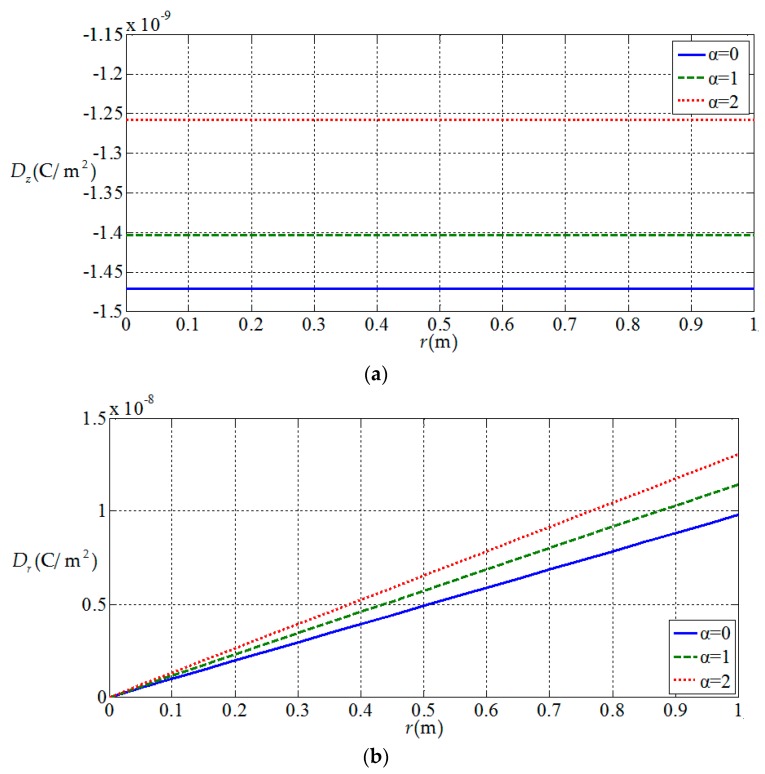
Variation of electric displacement with coordinates r at z=h/4. (**a**) Electric displacement $D_{z}(r,h/4)$ at z=h/4; (**b**) electric displacement $D_{r}(r,h/4)$ at z=h/4.

**Figure 13:**
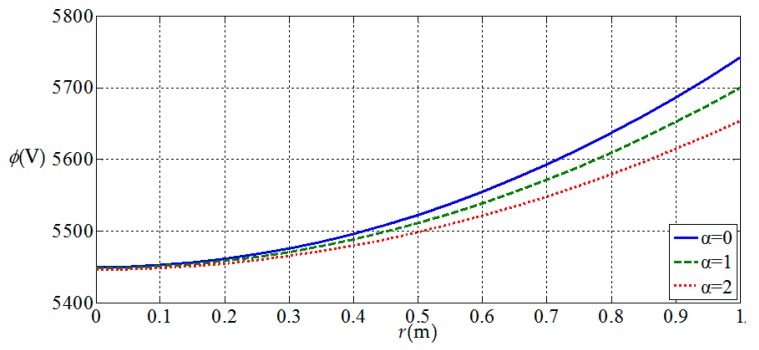
Variation of electric potential ϕ(r,h/4) with coordinates r at z=h/4.

Figure 10, Figure 11, Figure 12 and Figure 13 show the variation of the elastic displacement and stress, as well as the electric displacement and electric potential with the coordinate r at z=h/4. From Figure 10, it can be found that the elastic displacements change linearly along r direction, and they have almost no change with the increases of functional gradient parameter α. From Figure 11, Figure 12 and Figure 13, we can know that the variation curves of the elastic stress, electric displacement, and electric potential of the functionally graded piezoelectric circular plate (α≠0) are deviated from the uniform piezoelectric plate (α=0) and the degree of deviation increases with the increase of functional gradient parameter α, in which $D_{r}$ and ϕ increase from center to edge of the plate and $\sigma_{r}$ decreases along the same direction while $D_{z}$ and $\sigma_{z}$ remain unchanged from center to edge of the plate. In addition, $\sigma_{z}$, $D_{r}$, and $D_{z}$ change almost linearly along the r direction, and $\sigma_{r}$ and ϕ change nonlinearly along the r direction. These characteristics can be used as a reference for the analysis and design of functionally gradient piezoelectric plates.

## 4. Conclusions

In this study, the electroelastic solution of the axisymmetric deformation problem of functionally graded piezoelectric circular plates under the action of combined mechanical loads was derived by supposing the variable separation form of the displacement function and electrical potential function. Assuming that all the electroelastic materials parameters vary according to the same gradient function along the thickness direction, the electromechanical coupling effect of functionally graded piezoelectric circular plates under the combined mechanical loads was analyzed.

This work may be regarded as a theoretical reference for the analysis of functionally graded piezoelectric materials and structures. Specially, the solving method presented here can also be conveniently applied to other cases under the action of a single mechanical load or under different boundary conditions. Moreover, this work may be extended into the other problem under external electrical loads; in this case, the displacement function used for the solution needs to be modified to some extent. This work may also be extended to functionally graded beams and plates with different properties in tension and compression [36,37]. Obviously, the introduction of different moduli in tension and compression may bring some new issues, which will further complicate the solving of the problem. We will carry out these interesting works in the future. 

## Figures and Tables

**Table 1:** Material constants.

Property	Constants
Elastic(10^9^N/m^2^)	$c_{11}^{0}=c_{22}^{0}=74.1$, $c_{33}^{0}=83.6$, $c_{12}^{0}=45.2$, $c_{13}^{0}=c_{23}^{0}=39.3$, $c_{44}^{0}=c_{55}^{0}=13.17$, $c_{66}^{0}=14.45$
Piezoelectric(C/m^2^)	$e_{31}^{0}=e_{32}^{0}=-0.16$, $e_{33}^{0}=0.347$, $e_{15}^{0}=-0.138$, $e_{24}^{0}=0$
Dielectric(F/m)	$\lambda_{11}^{0}=\lambda_{22}^{0}=8.25\times 10^{-11}$, $\lambda_{33}^{0}=9.02\times 10^{-11}$

## Appendix A

Let us introduce two stress functions F(r,z) and ψ(r,z)

(A1) $$\sigma_{r}=F_{,zz}+r^{-1}\psi_{,r},\;\sigma_{\theta }=F_{,zz}+\psi_{,rr},\;\sigma_{z}=r^{-1}{\left(rF_{,r}\right)}_{,r},\;\tau_{rz}=-F_{,rz}.$$

In this way, the Equation (2) is satisfied automatically. Then, suppose that the stress functions have the following form [32]

(A2) $$F(r,z)=\sum_{i=0}^{n}r^{i}F_{i}(z),\;\psi (r,z)=\sum_{i=0}^{n}r^{i}\psi_{i}(z),\;(n=0,1,2\ldots ),$$

in which $F_{i}(z)$ and $\psi_{i}(z)$ are undetermined functions. Substituting Equation (A2) into Equation (A1), we can obtain

(A3) $$\begin{array}{l} \sigma_{z}=\frac{F_{1}}{r}+\sum_{i=0}^{n}{\left(i+2\right)}^{2}r^{i}F_{i+2},\;\sigma_{r}=\frac{\psi_{1}}{r}+\sum_{i=0}^{n}r^{i}[F_{i,zz}+(i+2)\psi_{i+2}],\\ \sigma_{\theta }=\sum_{i=0}^{n}r^{i}[F_{i,zz}+(i+2)(i+1)\psi_{i+2}],\;\tau_{rz}=-\sum_{i=0}^{n}(i+1)r^{i}F_{i+1,z},\;(n=0,1,2\ldots ) \end{array}.$$

The stress components $\sigma_{z}$ and $\sigma_{r}$ are limited values at r=0, thus we have

(A4) $$F_{1}(z)=0,\;\psi_{1}(z)=0.$$

From Equation (A4), we may infer that all the odd terms of r in Equation (A2) could be zero. From Equations (4) and (6) we can know the electric potential ∂ϕ/∂z corresponds to the stresses $\sigma_{z}$, $\sigma_{\theta }$, and $\sigma_{r}$. So, we can see that all the odd terms of r in the expression of electric potential ϕ are also equal to zero. Thus, the electric potential ϕ can be expressed as

(A5) $$\phi (r,z)=\sum_{i=0}^{n}r^{i}\phi_{i}(z),\;(n=0,2,4\ldots ).$$

Moreover, the Equation (4) can be transformed into

(A6) $$\begin{array}{l} \varepsilon_{r}=s_{11}\sigma_{r}+s_{12}\sigma_{\theta }+s_{13}\sigma_{z}+d_{31}E_{z}\\ \varepsilon_{\theta }=s_{12}\sigma_{r}+s_{11}\sigma_{\theta }+s_{13}\sigma_{z}+d_{31}E_{z}\\ \varepsilon_{z}=s_{13}\sigma_{r}+s_{13}\sigma_{\theta }+s_{33}\sigma_{z}+d_{33}E_{z} \end{array},$$

in which $s_{ij}$ are the flexibility coefficients and $d_{ij}$ are the piezoelectric constants. Substituting Equations (5), (6), and (A3) into (A6), we get

(A7) $$\begin{array}{l} \frac{u_{r}}{r}=s_{12}\sum_{i=0}^{n}r^{i}[F_{i,zz}+(i+2)\psi_{i+2}]+s_{11}\sum_{i=0}^{n}r^{i}[F_{i,zz}+(i+2)(i+1)\psi_{i+2}]\\ \;+s_{13}\sum_{i=0}^{n}{\left(i+2\right)}^{2}r^{i}F_{i+2}-d_{31}\sum_{i=0}^{n}r^{i}\frac{\partial \phi_{i}(z)}{\partial z},\;(n=0,2,4\ldots )\\ \frac{\partial u_{z}}{\partial z}=s_{13}\sum_{i=0}^{n}r^{i}[F_{i,zz}+(i+2)\psi_{i+2}]+s_{13}\sum_{i=0}^{n}r^{i}[F_{i,zz}+(i+2)(i+1)\psi_{i+2}]\\ \;+s_{33}\sum_{i=0}^{n}{\left(i+2\right)}^{2}r^{i}F_{i+2}-d_{33}\sum_{i=0}^{n}r^{i}\frac{\partial \phi_{i}(z)}{\partial z},\;(n=0,2,4\ldots ) \end{array}.$$

From Equation (A7), we can obtain

(A8) $$\begin{array}{l} u_{r}=s_{12}[\sum_{i=0}^{n}r^{i+1}[F_{i,zz}+(i+2)\psi_{i+2}]]+s_{11}[\sum_{i=0}^{n}r^{i+1}[F_{i,zz}+(i+2)(i+1)\psi_{i+2}]]\\ +s_{13}[\sum_{i=0}^{n}{\left(i+2\right)}^{2}r^{i+1}F_{i+2}]-d_{31}\sum_{i=0}^{n}r^{i+1}\frac{\partial \phi_{i}(z)}{\partial z},\;(n=0,2,4\ldots )\\ u_{z}=s_{13}[\sum_{i=0}^{n}r^{i}\int_{0}^{z}[F_{i,zz}+(i+2)\psi_{i+2}]dz]+s_{13}[\sum_{i=0}^{n}r^{i}\int_{0}^{z}[F_{i,zz}+(i+2)(i+1)\psi_{i+2}]dz]\\ +s_{33}[\sum_{i=0}^{n}{\left(i+2\right)}^{2}r^{i}\int_{0}^{z}F_{i+2}dz]-d_{33}\sum_{i=0}^{n}r^{i}\phi_{i}(z),\;(n=0,2,4\ldots ) \end{array}.$$

From Equation (A8) we can see that all the even items of r in the expression of $u_{r}$ are zero and all the odd items of r in the expression of $u_{z}$ are zero. Then, according to the boundary conditions of simply supported circular plates, we can finally get the forms of the displacement and the electric potential as follows (i.e., Equation (8); the more detailed derivation can be found in [32]):

(A9) $$\begin{array}{l} u_{r}(r,z)=ru_{1}(z)+r^{3}u_{3}(z)\\ u_{z}(r,z)=w_{0}(z)+r^{2}w_{2}(z)+r^{4}w_{4}(z)\\ \phi (r,z)=\phi_{0}(z)+r^{2}\phi_{2}(z)+r^{4}\phi_{4}(z) \end{array}\}.$$

In addition, similar expressions for displacement and potential function may be found in the analysis of functionally graded piezoelectric beams [34,35], in which the stress function U and the potential functions Φ were expressed in the form

(A10) $$\begin{array}{l} U(x,z)=\frac{x^{2}}{2}f(z)+xf_{1}(z)+f_{2}(z)\\ \Phi (x,z)=x^{2}f_{3}(z)+xf_{4}(z)+f_{5}(z) \end{array}\}.$$

in which *x* represents the longitudinal direction of the beam (similar to the radial direction *r* in the plate problem) and *z* stands for the thickness direction (similar to the transverse direction *z* in the plate problem). From the similarities of two sets of expression of beams and plates, we may find some consistencies in the analyses of beams and plates. For example, the derived analytical solutions both satisfy exactly the boundary conditions on the upper and lower surfaces of beams and plates (main boundary), while both satisfy approximately, in the Saint Venant sense, the end conditions for beams and the circumferential boundary conditions for plates (local boundary).
